# Retraction: Kuo, W.S. et al. Amelioration of Experimentally Induced Arthritis by Reducing Reactive Oxygen Species Production through the Intra-Articular Injection of Water-Soluble Fullerenol. *Nanomaterials* 2019, *9*, 909

**DOI:** 10.3390/nano10030417

**Published:** 2020-02-27

**Authors:** Wen-Shuo Kuo, Chia-Tse Weng, Jian-Hua Chen, Chao-Liang Wu, Ai-Li Shiau, Jeng-Long Hsieh, Edmund Cheung So, Po-Ting Wu, Shih-Yao Chen

**Affiliations:** 1 School of Chemistry and Materials Science, Nanjing University of Information Science and Technology, Nanjing 210044, China; wskuo88@gmail.com; 2 School of Environmental Science and Engineering, Nanjing University of Information Science and Technology, Nanjing 210044, China; 3 Center for Micro/Nano Science and Technology, National Cheng Kung University, Tainan 701, Taiwan; 4 Department of Internal Medicine, National Cheng Kung University Hospital, College of Medicine, National Cheng Kung University, Tainan 701, Taiwan; ctweng@mail.ncku.edu.tw; 5 Department of Anesthesia, An Nan Hospital, China Medical University, Tainan 709, Taiwan; aptx4869jfk@gmail.com; 6 Department of Anesthesia, China Medical University, Taichung 404, Taiwan; 7 Department of Biochemistry and Molecular Biology, College of Medicine, National Cheng Kung University, Tainan 701, Taiwan; wumolbio@mail.ncku.edu.tw; 8 Department of Microbiology and Immunology, College of Medicine, National Cheng Kung University, Tainan 701, Taiwan; alshiau@mail.ncku.edu.tw; 9 Department of Nursing, College of Nursing, Chung Hwa University of Medical Technology, Tainan 717, Taiwan; pipi58871053@yahoo.com.tw; 10 Graduate Institute of Medical Sciences, Chang Jung Christian, Tainan 711, Taiwan; 11 Department of Orthopedics, College of Medicine, National Cheng Kung University, Tainan 701, Taiwan; 12 Department of Orthopedics, National Cheng Kung University Hospital Dou-Liou Branch, College of Medicine, National Cheng Kung University, Yunlin 640, Taiwan; 13 Department of Orthopedics, National Cheng Kung University Hospital, College of Medicine, National Cheng Kung University, Tainan 701, Taiwan; 14 Department of Biomedical Engineering, National Cheng Kung University, Tainan 701, Taiwan; 15 Medical Device R & D Core Laboratory, National Cheng Kung University Hospital, Tainan 701, Taiwan

One of the contributors to the published paper [1] did not provide permission for the data presented to be published and we have therefore taken the decision to retract the paper. We apologize to readers of *Nanomaterials*. The decision to retract has been taken in agreement with the authors.

MDPI is a member of the Committee on Publication Ethics and takes the responsibility to uphold strict ethical policies and standards very seriously.
